# Comprehensive Management of Acute Ischemic Stroke in Psoriatic Patient

**DOI:** 10.1155/2023/6580971

**Published:** 2023-03-08

**Authors:** Al Rasyid, Taufik Mesiano, Mohammad Kurniawan, Rakhmad Hidayat, Reyhan Eddy Yunus, Endi Novianto, Ocktafiani Ocktafiani, Elvan Wiyarta, Salim Harris

**Affiliations:** ^1^Department of Neurology, Cipto Mangunkusumo Hospital, Faculty of Medicine, Universitas Indonesia, Jakarta 10430, Central Jakarta, Indonesia; ^2^Department of Radiology, Cipto Mangunkusumo Hospital, Faculty of Medicine, Universitas Indonesia, Jakarta 10430, Central Jakarta, Indonesia; ^3^Department of Dermatology and Venereology, Cipto Mangunkusumo Hospital, Faculty of Medicine, Universitas Indonesia, Jakarta 10430, Central Jakarta, Indonesia; ^4^Faculty of Medicine, Universitas Indonesia, Jakarta 10430, Central Jakarta, Indonesia

## Abstract

**Background:**

Stroke is one of the top causes of death and disability in several nations. Patients with psoriasis are susceptible to multiple comorbidities, including stroke. In addition to acute ischemic stroke, psoriasis and chronic inflammation require comprehensive treatment. Here, we present a comprehensive management case of a patient with an acute ischemic stroke and psoriasis. *Case Presentation*. A 42-year-old man came to the emergency department complaining of sudden left-sided weakness that started two and a half hours before being admitted to the hospital. The patient was treated with cyclosporine from 2013 to 2019 for a history of psoriasis. The patient was then treated for secondary stroke prevention using aspirin, vitamin B6, vitamin B12, folic acid, simvastatin, cyclosporine, and topical treatment. After two days of treatment, the patient's condition improved clinically, and he was discharged without further neurological deficits. As a home medication, the patient's cyclosporine was switched to the initial dose of methotrexate (7.5 mg/week) and titrated weekly to a response dose of 10 mg in the 10^th^ week. After three months of follow-up, the patient's condition remained stable, devoid of similar symptoms or sequelae.

**Conclusions:**

Cyclosporine should only be used for a maximum of 1 year for stroke management with psoriasis and be substituted for other systemic agents such as methotrexate. In addition, anticoagulants, antihypertensive, antihyperlipidemic, vitamin B6, vitamin 12, and folic acid regimens are highly recommended for comprehensive therapy of cardiovascular comorbidities.

## 1. Introduction

Stroke is one of the leading causes of disability and death in several countries. The incidence of stroke increased by 10% in developing countries within ten years (1990–2010) [1]. Ischemic stroke, the most common type, begins with a blockage of a blood vessel by a thrombus or embolism, which causes brain cells to experience metabolic disorders because they do not get a supply of blood, oxygen, and energy [1].

Psoriasis is a chronic disease mediated by the immune system, mainly characterized by thick, red, scaly patches. The prevalence of psoriasis varies from 0.14% in East Asia to 1.99% in Australia [2]. Psoriasis involves not only the skin but can also have a systemic impact. Approximately 75% of psoriasis patients have at least one comorbidity; some of them are related to chronic inflammatory processes such as uveitis, rheumatoid arthritis, and ulcerative colitis, and the worst is related to cerebrovascular diseases such as stroke [3]. The high prevalence of stroke in psoriasis can be due to the mechanism of this chronic inflammation. Therefore, comprehensive management is needed for an acute ischemic stroke patient with psoriasis.

## 2. Case Presentation

A 42-year-old man came to the emergency department complaining of sudden left-sided weakness that started two and a half hours before he was admitted to the hospital. The patient's face appears pulled to the right, but his speech remains unchanged. This is the first time the patient has felt such complaints. Complaints of headache, vomiting, seizures, double vision, visual disturbances, and decreased consciousness were denied.

The patient has a history of psoriasis and was treated with cyclosporine from 2013 to 2019. The patient did not suffer from hypertension, diabetes mellitus, stroke, or lung disease. The patient has smoked since 30 years ago, consumes alcohol, often eats fatty foods, and never exercises. There are no reports of the same complaint in the family. The patient is married and has two children.

On general physical examination, the patient was conscious with a blood pressure of 180/110. Skin examination revealed well-demarcatedplaque-shaped skin lesions on the face, chest, abdomen, arms, and legs. Neurological examination revealed a reactive pupil and central left facial nerve palsy. The motor strength of the upper extremities was 5555|2311 and that of the lower extremities was 5555|4411. Normal physiological reflexes with no pathological reflex, normal sensory function, and normal autonomic function were present.. The NIHSS (National Institutes of Health Stroke Scale) was five at admission, so the stroke code was activated.

**Figure 1:**
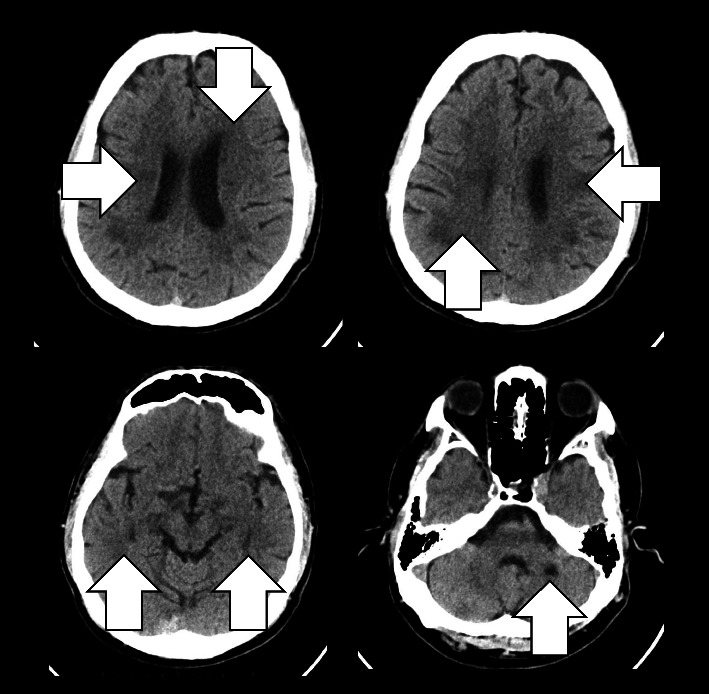
Solid arrows show multiple hypodense lesions of the white matter, which suggest an infarction on the CT brain.

Laboratory examination of the patient (Table 1) revealed leukocytosis with other results within normal limits. Noncontrast brain CT (Figure 1) shows ill-defined hypodensity of the periventricular and subcortical cerebral regions consistent with leukoaraiosis (small white arrows). Multiple chronic lacunar infarctions are visible in the pons, left cerebellum, right periventricular, and left subcortical regions (arrowheads). These findings may relate to chronic small vessel ischemic involvement. Areas of ill-defined hypodensities with sulcal effacement are noted in the right frontal and parietal lobes (large white arrows), suggesting the possibility of acute infarction with an ASPECT (Alberta Stroke Program Early CT Score) score of 8.

Based on clinical findings and investigations, the patient was diagnosed with acute ischemic stroke with left hemiparesis, left central facial nerve palsy, and psoriasis. The patient immediately underwent intravenous thrombolysis. After intravenous thrombolysis, blood pressure was 150/90 mmHg, and there were no complaints of headaches, nosebleeds, swollen lips, or bleeding gums. The NIHSS value after intravenous thrombolysis decreased to 2. After the intervention, the patient was transferred to the stroke unit for further comprehensive management.

While in the stroke unit, the risk factors for stroke were evaluated, and laboratory tests revealed uncontrolled hyperlipidaemia. The patient underwent transcranial and carotid doppler (TCD) and carotid doppler (CD), showing the bilateral intima-media thickness and normal accessible intracranial arteries. The patient was then managed with secondary stroke prevention using aspirin (1 × 80 mg), vitamin B6 (2 × 10 mg), vitamin B12 (2 × 50 mcg), folic acid (2 × 5 mg), and simvastatin (1 × 20 mg). The patient has been treated with cyclosporine (2 × 50 mg) and topical therapy for the skin (Liquor Carbonic Distillate (LCD) 3% in oleum cocos, LCD 5% in vaseline album, desoximetasone 0.35% ointment, fluocinolone acetonide 0.25% cream, and 5% salicylic acid in vaseline album).

After two days of treatment, the patient showed clinical improvement. The patient's hemodynamics were stable, and no neurological deficits were found, either motor, neurologic, or autonomic. As a home medication, the patient's cyclosporine was switched to the initial dose of methotrexate (7.5 mg/week) and titrated weekly to a response dose of 10 mg in the 10^th^ week. After three months, the patient's condition remained stable, without similar symptoms or sequelae.

## 3. Discussion

Managing stroke patients with psoriasis requires a fast, appropriate, and comprehensive approach. For stroke management, it was decided to do intravenous thrombolysis with alteplase at a dose of 0.6 mg/kg BW. The PRISM study on patients with mild symptomatic stroke showed that intravenous thrombolysis did not provide a better outcome than antiplatelet administration [4]. With an ASPECT score of 4, reperfusion with endovascular thrombectomy is also not an option. Based on the guidelines for managing acute ischemic stroke based on the 2019 AHA/ASA, thrombectomy was performed in patients with NIHSS and ASPECT scores higher than 6. The study shows that high ASPECT scores in patients with early onset are associated with the incidence of intracranial bleeding after endovascular thrombectomy [5].

The patient was then treated with aspirin for secondary prevention of stroke. Aspirin is recommended in patients with acute ischemic stroke within 24 to 48 hours after onset [6]. In individuals with mild stroke, 21 days of dual antiplatelet medication (aspirin and those began within 24 hours) may help prevent early secondary stroke for up to 90 days from the onset of symptoms. Dual antiplatelet therapy with aspirin (75 mg followed by 75 mg daily for 21 days) and clopidogrel (300 mg followed by 75 mg daily for 90 days) may be administered in this patient [6].

Vitamin B6, B12, and folic acid were also administered to the patient as secondary stroke prevention. Giving folic acid and B vitamins helps reduce homocysteine levels, which are currently known risk factors for stroke [7]. Simvastatin was also administered to this patient following the SPARCL trial, which discovered the benefits of statin therapy in lowering the risk of stroke [8]. Because hypertension is the most severe risk factor, blood pressure must be maintained in every stroke patient who has hypertension. Amlodipine, a calcium channel blocker, was prescribed as an antihypertensive medication for this patient.

Psoriasis care must be prioritized alongside stroke management because it is associated with cardiovascular complications. This control of chronic inflammation is the difference between treating stroke patients with psoriasis and those without psoriasis. Systemic inflammation in psoriasis can lead to endothelial dysfunction and atherosclerosis, leading to stroke due to the occlusion of blood vessels [9]. More significant inflammation in psoriasis with higher severity will increase endothelial dysfunction and lead to a greater risk of stroke [9]. Adequate treatment is needed in psoriasis to reduce vascular comorbidities.

On the other hand, the patient also received cyclosporine (2 × 50 mg) and topical therapy. Cyclosporine is an option for antipsoriasis therapy [9]. However, treatment with cyclosporine can cause impaired renal function, hypertension, and increased cardiovascular risk [9]. In addition, hyperlipidemia may occur in psoriasis patients treated with cyclosporine [9]. Therefore, we recommend that in patients with psoriasis, cyclosporine should only be used for a maximum of 1 year [10] and be substituted for other systemic agents, such as methotrexate [11].

In this case, the patient received an initial dose of methotrexate (7.5 mg/week) as home medication. Apart from the side effects, the patient's replacement therapy from cyclosporin to methotrexate was chosen because of a history of using cyclosporine for six years. The dose was also titrated to a dose response of 10 mg in the 10th week. Another study on the use of methotrexate in rheumatoid arthritis patients with stroke also showed that doses >8.5 mg/week were able to reduce the risk of acute ischemic stroke [12].

## 4. Conclusion

Cyclosporine should only be used for a maximum of 1 year for stroke management with psoriasis and be substituted for other systemic agents, such as methotrexate. In addition, to provide comprehensive management of cardiovascular comorbidities, the use of anticoagulant, antihypertensive, antihyperlipidemic, vitamin B6, vitamin 12, and folic acid regimens is highly recommended.

## Figures and Tables

**Table 1:** Patient's laboratory tests on admission.

Tests	Normal range	Result
Haemoglobin	13–17 g/dL	15.6 g/dL
Hematocrit	40–50%	44.3%
Leukocyte count	4.000–10.000 cells/*μ*L	10,900 cells/*μ*L
Platelet count	150.000–410.000 cells/*μ*L	238,000 cells/*μ*L
Blood glucose	60–140 mg/dL	133 mg/dL
PT	9.8–12.6 s	11.6 s
APTT	31–47 s	34.2 s
Natrium	136–145 mEq/L	139 mEq/L
Kalium	3.5–5.1 mEq/L	3.5 mEq/L
Chloride	98–107 mEq/L	105.5 mEq/L
Ureum	15–40 mg/dL	17.9 mg/dL
Creatinine	0.6–1.0 mg/dL	0.8 mg/dL
eGFR	93–139 mL/min/1.73 m^2^	107.1 mL/min/1.73 m^2^
AST	5–34 IU/L	34 IU/L
ALT	6–37 IU/L	42 IU/L

ALT: alanine aminotransferase; APTT: activated partial thromboplastin time; AST: aspartate aminotransferase; eGFR: estimated glomerular filtration rate; IU: international unit; PT: prothrombin time.

## Data Availability

No data were used to support this study.

## Abbreviations

AHA: American Heart Association
ASA: American Stroke Association
ASPECT: Alberta Stroke Program Early CT Score
BW: Body weight
CD: Carotid doppler
CHANCE: Clopidogrel in high-risk patients with acute nondisabling cerebrovascular events
CT: Computed tomography
LCD: Liquor carbonis distillate
NIHSS: National Institutes of Health Stroke Scale
PRISMS: The Parkinson's real-world impact assessment
SPARCL: Stroke prevention by aggressive reduction in cholesterol levels
TCD: Transcranial doppler.
